# Disseminated *Aspergillus citrinoterreus* and concurrent localized dermal phaeohyphomycosis in an immunosuppressed dog

**DOI:** 10.1002/ccr3.7573

**Published:** 2024-02-22

**Authors:** David Sender, Benjamin Hulsey, Connie Cañete‐Gibas, Nathan Wiederhold, Jung Keun Lee, Abigail Finley, Catherine Cruz, Mary E. White

**Affiliations:** ^1^ Department of Clinical Sciences, College of Veterinary Medicine Auburn University Auburn Alabama USA; ^2^ Department of Clinical Medicine, Midwestern College of Veterinary Medicine Midwestern University Glendale Arizona USA; ^3^ Department of Pathology and Laboratory Medicine University of Texas Health Science Center at San Antonio, University of Texas San Antonio Texas USA; ^4^ Department of Pathology, Midwestern College of Veterinary Medicine Midwestern University Glendale Arizona USA

**Keywords:** canid, emergency medicine, infectious diseases, veterinary

## Abstract

**Key Clinical message:**

We report on a dog with immune‐mediated hemolytic anemia (IMHA) treated with immunomodulatory therapy that developed phaeohyphomycosis and *Aspergillus citrinoterreus* infections. This is the first reported case of *A. citrinoterreus* in dogs. It details cytological and microbiological findings leading to diagnosis and highlights the importance of investigating new lesions in immunocompromised patients.

**Abstract:**

A 5‐year‐old Staffordshire terrier mix treated with immunosuppressive therapy for IMHA was diagnosed with concurrent disseminated *A. citrinoterreus* and localized *Curvularia lunata* infections. This case highlights the potential development of multiple concurrent opportunistic fungal infections and is the first reported case of *A. citrinoterreus* infection in a dog.

## Graphical abstract

Cytopictograph images demonstrating hyphael morphology differences between Aspergillus citrinoterrus (A) and *Curvularia lunata* (B).

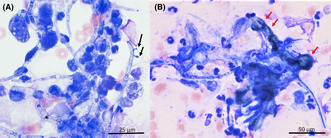

## 1 CASE PRESENTATION

A 5‐year‐old, male neutered, American Staffordshire mix presented to the Midwestern University Companion Animal Clinic (MWU CAC) for evaluation of an acute development of labored breathing after having been previously diagnosed with presumptive immune‐mediated hemolytic anemia (IMHA) that was treated with multimodal immunosuppressive therapy. The presumptive diagnosis of IMHA was made at MWU CAC a month prior to the current presentation according to the previously published consensus statement based on the presence of severe regenerative anemia (hematocrit 19%; RI, 36–60) with spherocytosis and a positive saline agglutination test as well as hyperbilirubinemia.^1^

The patient's work up on his initial presentation 1 month prior to presentation for respiratory distress included a complete blood count (CBC), serum biochemistry, urinalysis, thoracic radiographs, abdominal radiographs, abdominal ultrasound, fecal evaluation, and SNAP 4Dx test (IDEXX Laboratories, Inc.). Those results are described below:

CBC abnormalities showed a non‐regenerative, normochromic, normocytic anemia with a hematocrit of 20% (RI 36–60) and a hemoglobin of 6.4 g/dL (12.1–20.3), a neutrophilia of 10,842/μL (RI 2060–10,600) with band cells present at 139/μL (RI 0–300), and a mild monocytosis of 1251/μL (RI 0–840). Serum biochemical abnormalities included a moderate hyperbilirubinemia of 1.8 mg/dL (RI 0.1–0.6), mild hyperglycemia of 116 mg/dL (RI 60–110), mild hypokalemia of 3.5 mmol/L (RI 3.7–5.8). Urinalysis abnormalities included decreased urine specific gravity of 1.013 (RI 1.015–1.05), alkaline pH of 8 (RI 5.5–7), and struvite crystals 0–1 per high powered field (RI 0–0). Thoracic and abdominal radiographs reviewed by a board certified radiologist reported no clinical abnormalities. Abdominal ultrasound performed by a board certified internist was also clinically unremarkable with no significant abnormalities noted. Fecal evaluation was negative for intestinal parasitism. SNAP 4Dx was negative for *Dirofilaria immitis* antigen, antibody to *Anaplasma phagocytophilum*, antibody to *Anaplasma platys*, antibody to *Borrelia burgdorferi*, antibody to *Ehrlichia canis*, and antibody to *Ehrlichia ewingii*.

Further diagnostics during the month after initial presentation included *Babesia* polymerase chain reaction (PCR) (Protatek) as a potential cause of associative IMHA, as well as repeated CBCs and serum chemistries for monitoring of patient response to therapy. *Babesia* PCR was negative for *Babesia* genetic material. The IMHA was presumed to be nonassociative based on this work up that had been performed. However, a complete work up according to consensus guidelines to more definitively rule out associative IMHA was not performed. The patient had never traveled outside the state of Arizona.

Medications initially prescribed for treatment of IMHA included oral cyclosporine (Atopica. Greenfield, IN: Elanco US Inc.) at 5.4 mg/kg twice daily, oral prednisone at 1.5 mg/kg once daily, and oral clopidogrel at 1.0 mg/kg once daily. Two weeks later, the dosage of prednisone had been decreased to 1 mg/kg daily due to a stable hematocrit and concerns for medication side effects. Nine days after that, oral clindamycin therapy was started at 12.3 mg/kg twice daily due to an acute onset of paw swelling and self‐trauma to digits 3 and 4 of the right manus. Based on gross evaluation of the digits during physical exam, a presumptive diagnosis for the digital lesions was given to opportunistic bacterial infection secondary to immunosuppression. No abnormalities with his digits had been noted on any examination prior to that time. Five days later oral enrofloxacin therapy at 11.1 mg/kg once daily was initiated due to clinical progression of the digit lesions while a culture of the lesion was pending.

**FIGURE 1:**
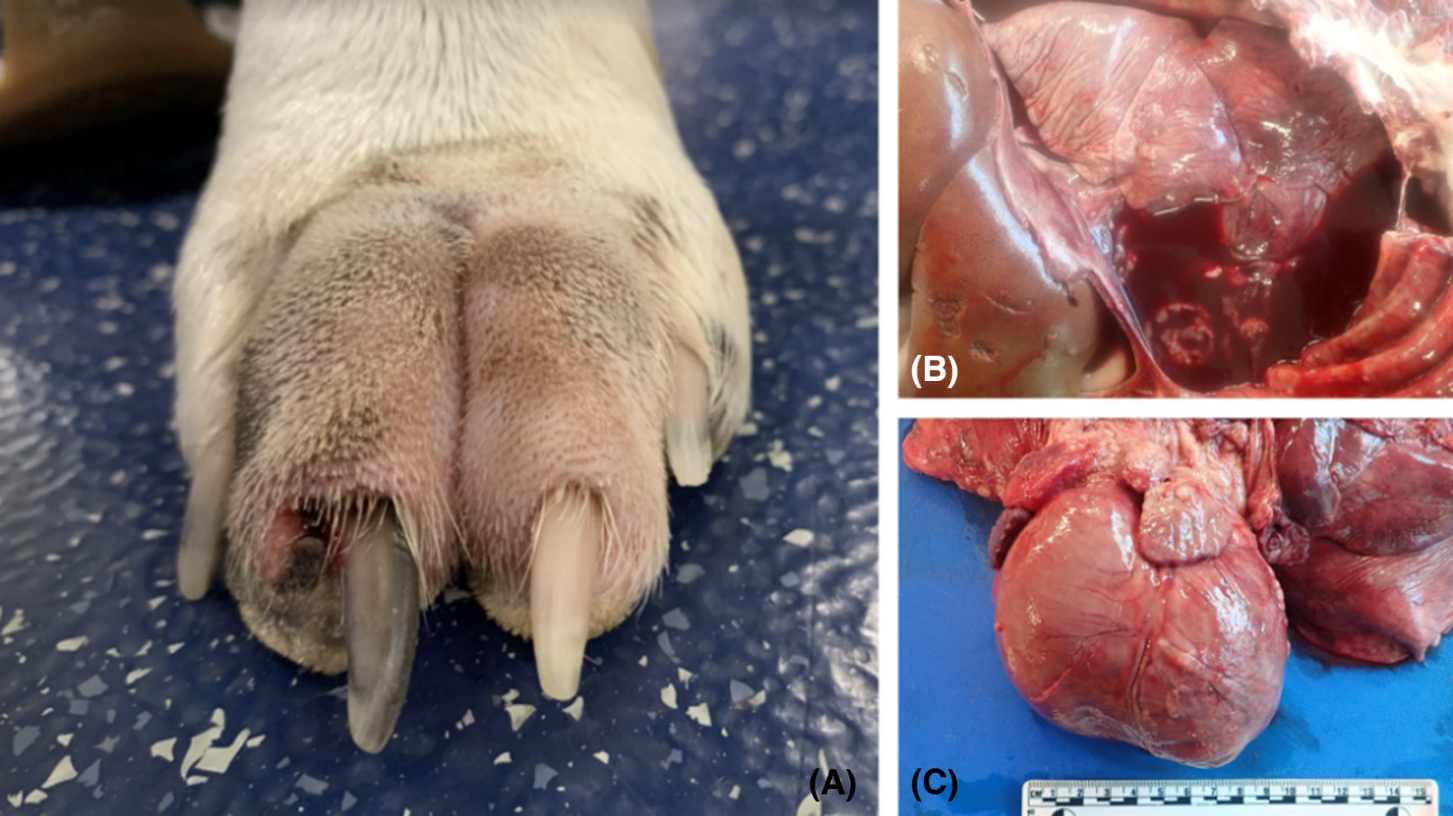
(A) Digits 3 and 4 of the right forelimb shows an open lesion and swelling of both digits. Localized *Curvularia* infection was suspected to have been acquired traumatically as is most common in veterinary patients. (B) Thoracic cavity on necropsy contains approximately 2 L of serosanguinous fluid. The liver (left) is pale and friable with an increased lobular pattern. (C) Heart and pericardium on necropsy examination. The epicardium of ventricles and auricles were multifocally thickened and irregular with fibrous material and white to tan pyogranulomatous nodules.

At the time of presentation for respiratory distress, results of physical examination included a rectal temperature of 101.4°F, heart rate of 140 beats per minute, resting respiratory rate of 80 breaths per minute, body condition score of 7/9, and quiet to dull mentation. Increased respiratory effort, moderate labored breathing, and muffled heart sounds were also noted. Pain was elicited on palpation of the right manus, which was edematous and erythematous throughout, with open ulceration of digit 3 and mild serosanguineous discharge from nail beds of digits 3 and 4 (Figure 1).

A CBC (Advia 2120i Hematology System) performed 24 h prior to presentation for respiratory distress revealed the following abnormalities: a hematocrit of 33% (RI, 36–60), platelet count of 338 K/μL (RI, 170–400), an increased mean cell volume of 82 fL (RI, 58–79), a reticulocytosis of 188 K/μL (RI, 0–0), a leukocytosis of 37.4 K/μL (RI, 4–15.5) characterized by a marked total neutrophilia of 29,546/μL (RI, 2060‐10,600) with a left shift (bands 5984/μL, RI, 0–300). No spherocytes were reported at that time. Serum biochemistry profile (Beckman Coulter AU480) performed 48 h prior to presentation for respiratory distress showed the following abnormalities: increased alanine transferase of 531 IU/L (RI, 12–118), alkaline phosphatase of 4813 IU/L (RI, 5–131), gamma‐glutamyl transferase of 212 IU/L (RI, 1–12), a mild hyperbilirubinemia of 0.7 mg/dL (RI, 0.1–0.3), and a mild total hypocalcemia of 8.6 mg/dL (RI, 8.9–11.4). The mild hyperbilirubinemia along with continued reticulocytosis was suggestive of ongoing low‐grade hemolysis and/or a component of blood loss (with gastrointestinal hemorrhage being the leading concern) and cholestasis associated with primary liver disease and/or sepsis.

**FIGURE 2:**
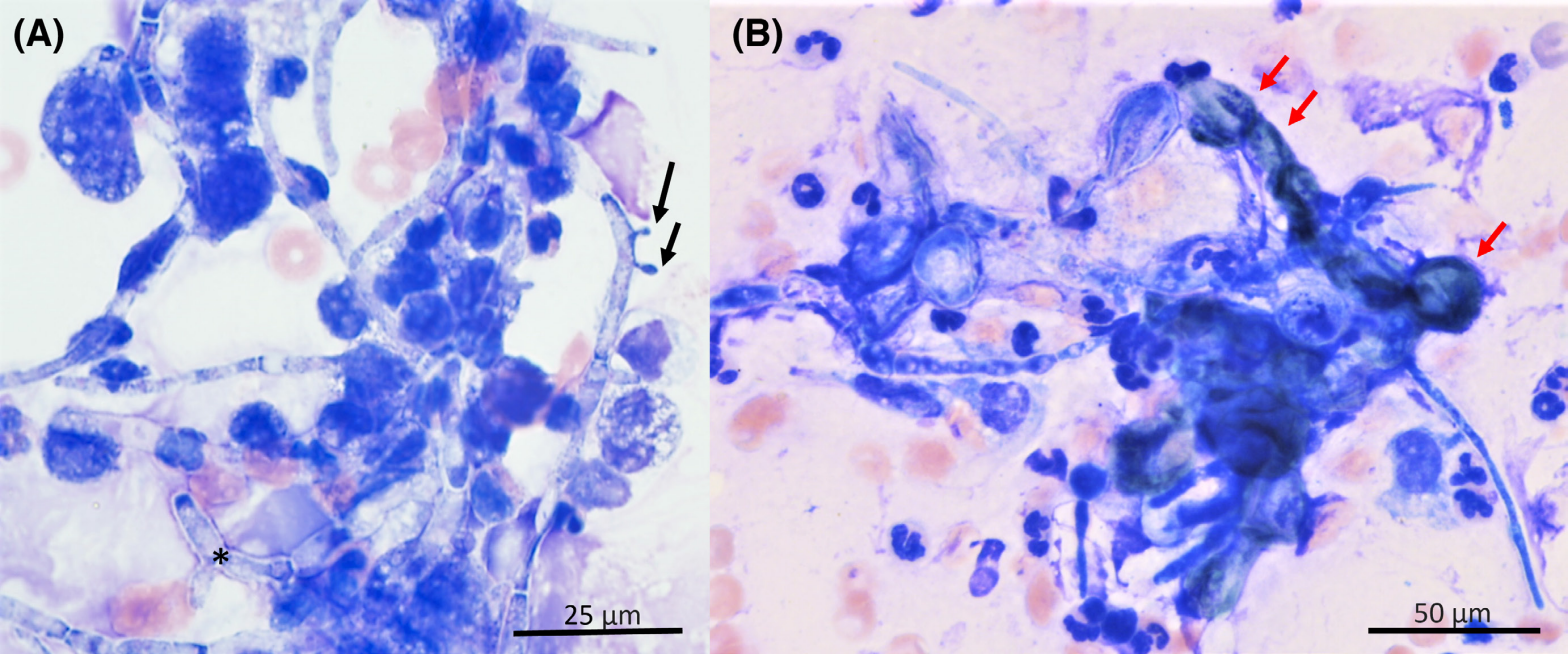
(A) Cytopictograph from pericardial effusion. The fungal hyphae exhibited the following morphologic characteristics: irregular septations that vary in length (10–28 μm) and width (1–4 μm); pale to moderately basophilic staining with varying amounts of fine eosinophilic granulation; acute and perpendicular branching of hyphae (asterisk); and presence of occasional suspect accessory conidia (black arrows). Wright Giemsa, 100×. B) Cytopictograph from fine needle aspirate of digit 3: The hyphae exhibit the following morphologic characteristics: irregular septations that vary in length (10–40 μm) and width (1–6 μm), moderate to deeply basophilic staining; acute and perpendicular branching and exhibit large, round, bulbous ends that contain irregularly distributed fine green to black pigmentation (red arrows). Wright Giemsa, 50×.

Thoracic point of care ultrasound revealed bilateral, severe pleural effusion and pericardial effusion. Pleural fluid was collected via bilateral ultrasound‐guided thoracocentesis. Approximately 861 mL of serosanguinous, slightly viscous, fluid with occasional, grossly apparent, free floating, tan, friable material. The total protein of the pleural effusion was 2.2 g/dL. Cell counts of the effusion were not performed, but direct smears were created from the pleural and pericardial fluid, as well as squash preparations of the previously described free floating material seen in the pleural fluid. Cytologic specimens of the pleural and pericardial fluid evaluated by a board‐certified clinical pathologist showed numerous degenerate to poorly preserved neutrophils. There were moderate numbers of moderately vacuolated macrophages that occasionally contained phagocytized cellular debris. Both the pericardial and pleural fluid contained moderate numbers of extracellular fungal hyphae (Figure 2A) that were often surrounded by degenerate neutrophils, fewer macrophages, and proteinaceous streaming debris. The majority of the hyphal structures exhibited the following morphologic characteristics: irregular septations that varied in size, with the length ranging from 10 to 28 μm and width ranging from 1 to 4 μm; pale to moderately basophilic staining with varying amounts of fine eosinophilic granulation; acute and perpendicular branching of hyphae; and the presence of few, small structures that were composed of a single, round to oval structure with a narrow stalk branching off central cells (suspect accessory conidia). There were rare to occasional hyphae that exhibited short, cuboidal to rounded, regular septation that was interpreted as a continuum of the previously described fungal population. Both fluid samples were interpreted as marked pyogranulomatous inflammation with intralesional septate, fungal hyphae.

Cytologic smears of the discharge and fine needle aspirates from digits 3 and 4 were evaluated by a board‐certified clinical pathologist. Nucleated cells were found individually and in intermediate to large aggregates that were often associated with extracellular hyphal structures. The majority of the hyphae were elongated with tapering ends, occasional acute branching, and partitioned by irregular septations that varied in size, with the length ranging from 10 to 40 μm and width ranging from 1 to 6 μm. Rarely the hyphae exhibited large, round, bulbous ends that contained, irregularly distributed, fine green to black pigmentation (Figure 2B). The predominant cell population consisted of numerous degenerate neutrophils and fewer moderate to markedly vacuolated macrophages that occasionally exhibited leukophagia and erythrophagia. There were occasional multinucleated giant cells seen on scanning. The digit cytology was interpreted as marked pyogranulomatous inflammation with intralesional dematiaceous fungal hyphae. Due to the difference of fungal morphologies between lesions of the digit and thoracic cavity, concern for a mixed fungal infection was suspected, and culture and PCR were recommended for further evaluation.

**FIGURE 3:**
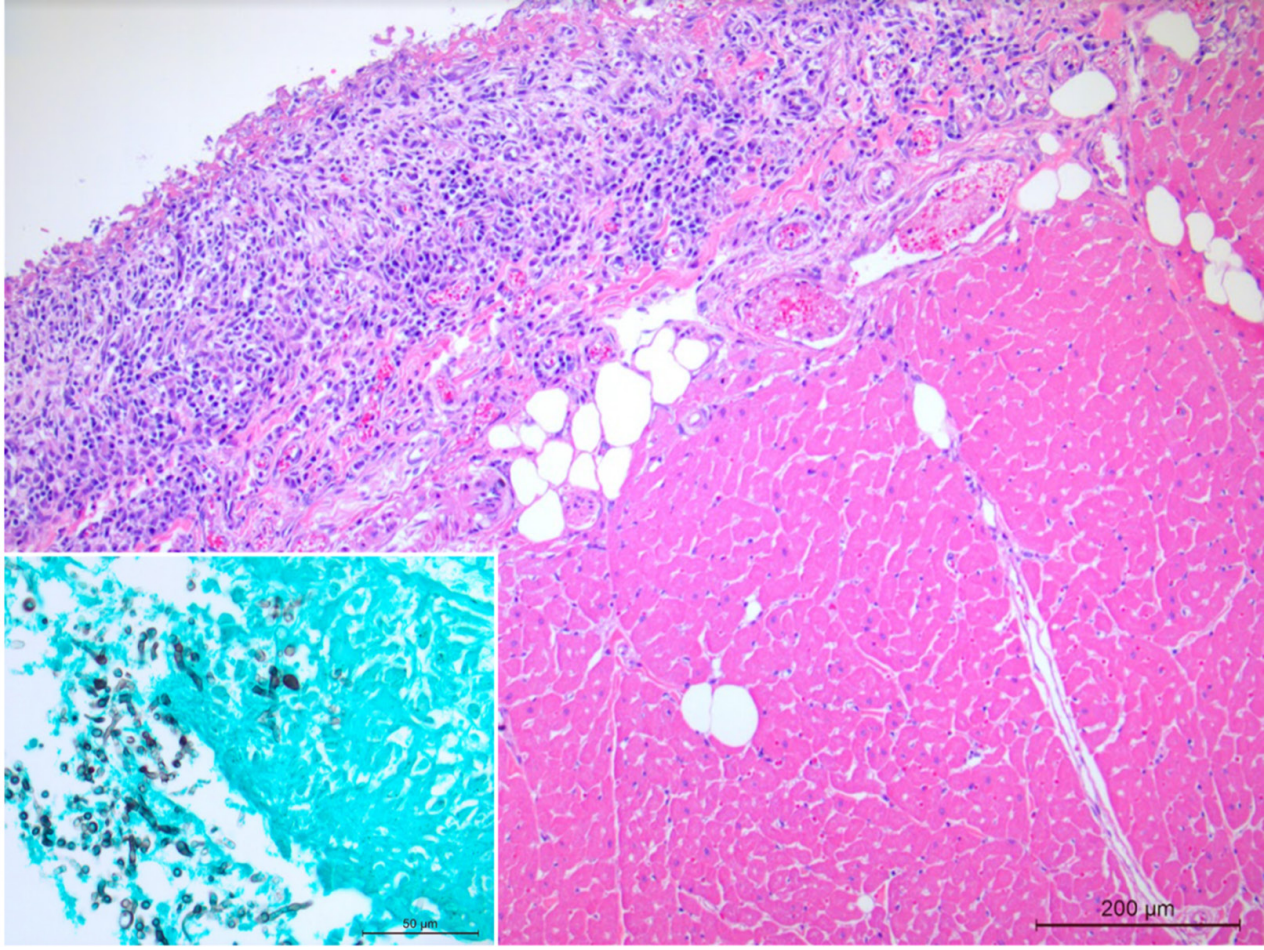
Heart: The pericardium is markedly thickened with severe diffuse pyogranulomatous inflammation with multiple GMS (Grocott's Methenamine silver stain)‐positive fungal hyphae (insert: *Aspergillus citrinoterreus*).

Given the poor prognosis, the owner elected palliative care, and euthanasia was performed the following day after respiratory signs recrudesced. A necropsy was performed, and histopathology was reviewed by a board‐certified anatomic pathologist. During gross examination, liver, kidney, lung, pleural and pericardial fluid, and nail bed specimens were submitted for fungal culture and mycologic assessment. The thoracic cavity contained approximately 2 L of serosanguineous fluid (Figure 2B). Affecting approximately 80%, the pulmonary pleura and mediastinum were markedly thickened with multifocal to coalescing, white‐tan, firm plaques and nodules. The pericardium was diffusely and markedly thickened with a pyogranulomatous inflammation and contained approximately 110 mL of red serosanguineous fluid (Figures 2C and 3). The epicardium was also multifocally thickened, rough, and irregular with the same white to tan nodules (Figure 2C). Examination of the abdomen showed multiple, fairly demarcated, white, firm nodules ranging from 0.1 to 0.3 cm in diameter and affecting approximately 5% of the renal cortices bilaterally. Examination of the right, distal forelimb revealed markedly edematous, firm, red digits 3 and 4. An open 1.0 × 0.6 cm wound was noted on the medial aspect of the right front 3rd digit with pink, purulent exudation. There was a small amount of tissue loss around the nail bed of the right front 4th digit with a small amount of yellow‐white fluid (Figure 2A).

Significant histopathologic findings included severe, fibrinonecrotizing, pyogranulomatous dermatitis with intralesional and intravascular, brown‐pigmented septate fungal hyphae and conidia, necrotizing vasculitis, and fibroplasia of the nail beds of digits 3 and 4 of the right forelimb. Similar severe, fibrinonecrotizing, pyogranulomatous pneumonia, pleuritis, pericarditis, and nephritis with intralesional fungal hyphae that exhibited different morphology from hyphae seen on digits 3 and 4, fibroplasia, and serosanguineous effusion were also noted (Figures 1 and 2). Fungal cultures were obtained from specimen taken from the visceral organs (lung, liver, and kidney) and presumptively identified as *Aspergillus terreus* by matrix‐assisted laser desorption ionization time‐of‐flight (MALDI‐ToF MS). Another culture of a dematiaceous fungus was isolated from nail beds of digits 3 and 4 of the right front limb and was identified by MALDI‐ToF MS as *Curvularia lunata*. The MALDI‐ToF filamentous fungal library used for identification at MWU College of Veterinary Medicine Clinical Microbiology Laboratory was validated using Bruker's MALDI Biotyper Standard Operating Procedure for Cultivating and Sample preparation for Filamentous Fungi using the following fungal strains: *Aspergillus fumigatus* ATCC 204305 (BSL2), *Aspergillus niger* ATCC 16888 (BSL1), *Fusarium solani* ATCC 36031(BSL2), *Microsporum canis* ATCC 36299 (BSL2), *Trichophyton rubrum* ATCC 28188 (BSL2). All strains had MALDI scores ≥2.0. The score for the presumed *Aspergillus terreus* was 2.24, and the *C. lunata* score was 2.06. A culture slant of the presumptive *Aspergillus terreus* UTHSCSA DI20‐341 isolate obtained from the lung was submitted for species confirmation to the Fungus Testing Laboratory at the University of Texas Health Science Center at San Antonio (FTL).

## 2 MYCOLOGICAL METHODS AND RESULTS

### 2.1 Morphology and phenotypic assessment

**FIGURE 4:**
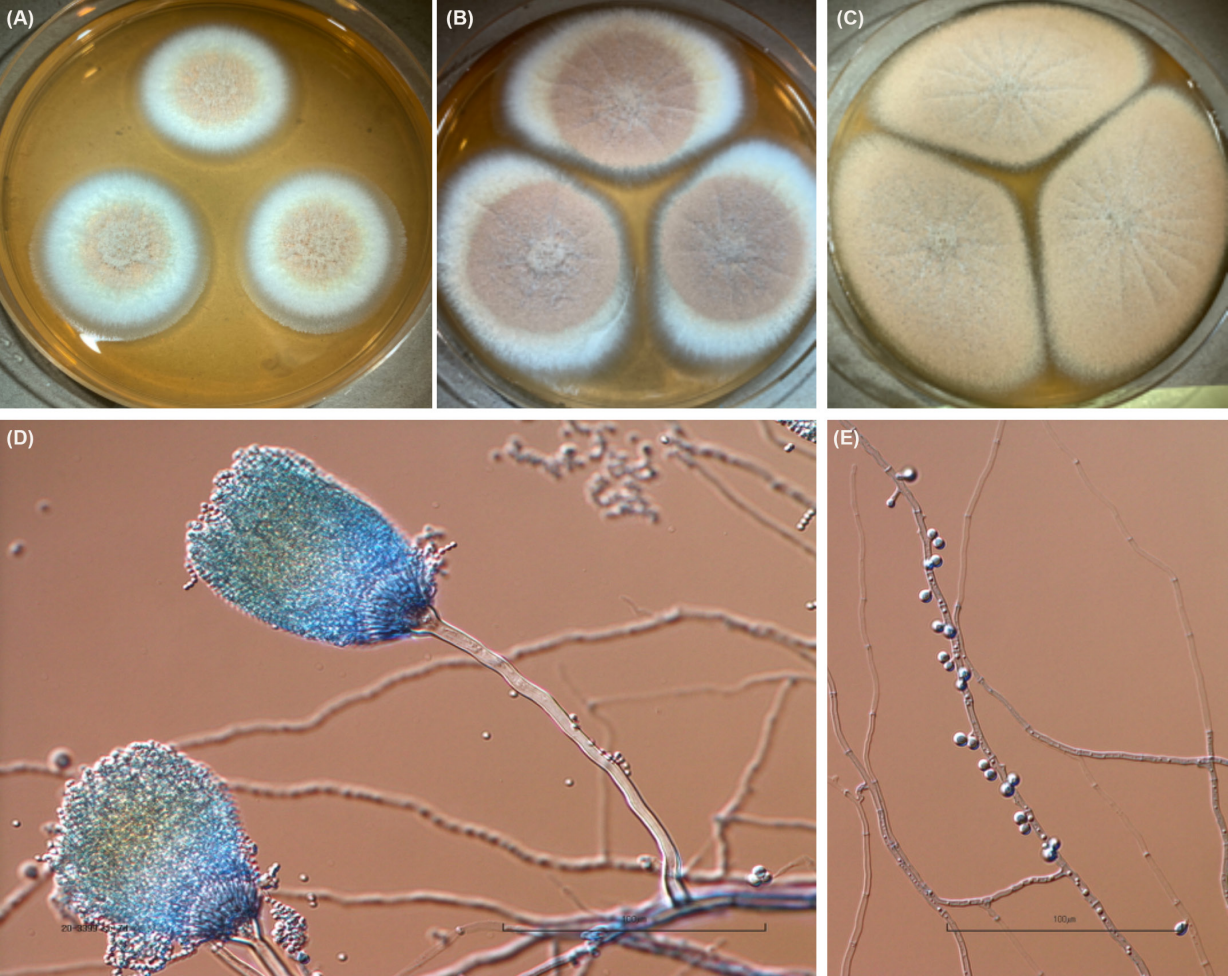
*Aspergillus citrinoterreus* (UTHSCSA DI20‐341). (A–C) Colonies on MEA at 37°C at 7, 25, and 30 days, respectively. (D,E) Light micrographs of vesicles and accessory conidia at 7 days.

Isolate UTHSCSA DI20‐341 was three‐point inoculated onto Creatinine agar (Crea), Czapek yeast extract agar (CYA), malt extract agar (MEA), yeast extract sucrose agar (YES), and potato flakes agar (PFA) and were incubated in the dark at 25°C for 7 days. Growth was also assessed at 30, 37, 45, and 50°C. Slide cultures were set up and were mounted on lactophenol cotton blue after 7 days. Morphological and microscopic characteristics typical of members of the *Aspergillus* section Terrei, including *Aspergillus citrinoterreus* showed cinnamon‐colored colonies on MEA at 7 days at 25, 30, and 37°C (Figure 4A–C) with intense yellow diffusing pigment, globose to subglobose yellowish conidia, and smaller obovoid accessory conidia on short stalks (Figure 4D,E). Growth at 25, 30, 37, and 45°C were observed but not at 50°C. Sporulation was abundant on MEA compared to the other culture media. It is important to note, that these characteristics are also exhibited by other species in the *Aspergillus* section Terrei with subtle differences that are difficult to distinguish between.

### 2.2 DNA sequencing and phylogenetic analysis

Genomic DNA was extracted from harvested mycelia on PFA and sequenced using Bt2a and Bt2b primers for the partial beta‐tubulin (*BenA*) gene region and CF1 and CF4 primers for the partial calmodulin (*CaM*) gene region as previously described.^2^, ^3^, ^4^ Newly generated sequences were deposited in the GenBank database under the accession numbers MW419109 (*BenA*), MW419108 (*CaM*). BLASTn searches were conducted for presumptive species identification. BLASTn results for *BenA* and *caM* showed 99%–100% identity with *A. citrinoterreus* sequences in GenBank. Many of the *A. citrinoterreus* sequences in GenBank were identified as *Aspergillus terreus* but were reidentified later as *A. citrinoterreus*.^5^

**FIGURE 5:**
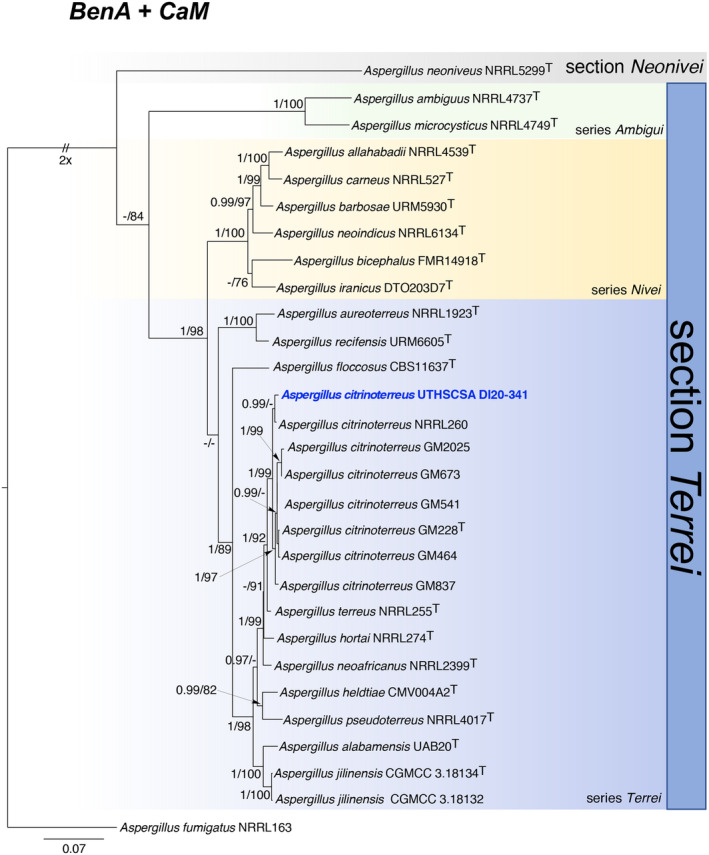
A maximum likelihood tree constructed from combined sequences of partial *BenA* and *CaM* showing the relationship of isolate UTHSCSA DI20‐341 with representative species in the *Aspergillus* section Terrei. Values at the nodes are Bayesian posterior probability (BI) ≥ 0.95 (right) and bootstrap (BS) ≥ 70% (left). Ex‐type cultures are marked with a ^T^. Branches with double‐bars are truncated two‐fold.

**FIGURE 6:**
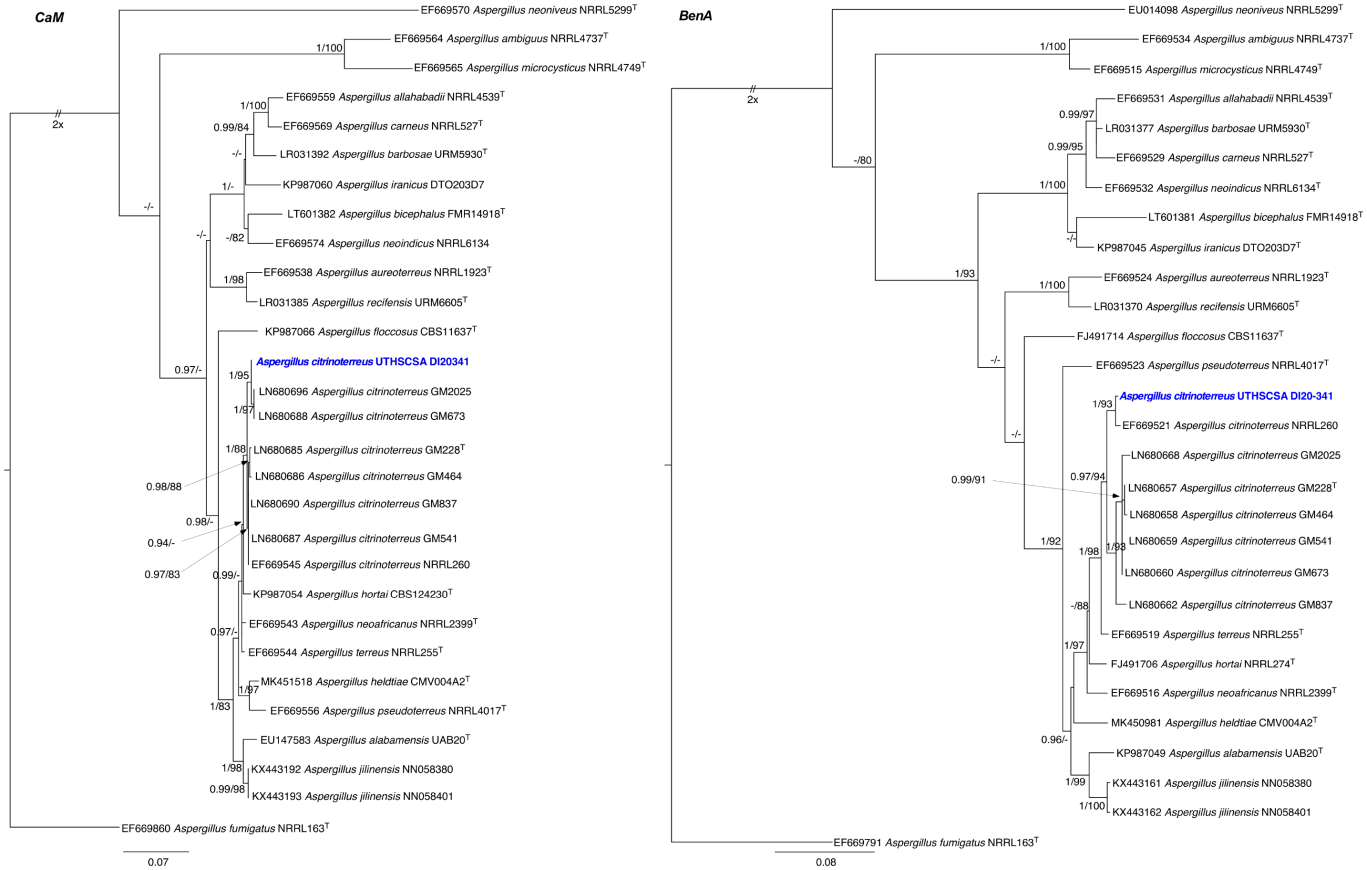
Maximum likelihood trees from sequences of individual genes *BenA* and *CaM* showing the relationship of isolate UTHSCSA DI20‐341 with representative species in the *Aspergillus* section *Terrei*. Values at the nodes are Bayesian posterior probability (BI) ≥ 0.95 (right) and bootstrap (BS) ≥ 70% (left). Ex‐type cultures are marked with a ^T^. Branches with double‐bars are truncated two‐fold.

Based on BLASTn results, the dataset was compiled using the newly generated sequences and publicly available representative sequences of authentic and type strains of species in the *Aspergillus* section *Terrei*. Maximum likelihood trees were generated on individual locus and combined using the IQ‐Tree.^6^ The best fitting model for each locus and combined as determined by corrected Akaike Information Criterion as implemented in IQ‐TREE was TIM3e + G4 (*BenA*), TNe + I + G4 (*CaM*), and TNe + I + G4 (*BenA* + *CaM*). Branch supports were estimated by 1000 bootstrap replicates (BS) and Bayesian inference (BI) with MrBayes v.3.2.6 using a Markov chain Monte Carlo (MCMC) algorithm.^7^, ^8^ BI analyses were run for 2 × 10^6^ generations, sampling every 100 replicates until convergence (standard deviation of split frequency) of <0.01. The first 25% trees were discarded as burnin and the remaining were combined into one tree with 50% majority rule consensus and visualized in FigTree v1.4.4 (http://tree.bio.ed.ac.uk/software/figtree/). A threshold of ≥70% was used as a cutoff for bootstrap significantly supported nodes and BI posterior probabilities above 0.95 were considered significant. *Aspergillus fumigatus* NRRL 163^T^ was used as the outgroup species. Following the genealogical concordance phylogenetic species recognition (GCPSR), results of the phylogenetic analyses of the combined loci (Figure 5) and each individual locus (Figure 6) showed the isolate consistently grouping with the type and other strains of *A. citrinoterreus* in each of the gene tree and confirmed the identity of the isolate as *A. citrinoterreus* with high branch support (*CaM* = BI/BS = 1.00/88%; *BenA* = BI/BS 0.97/ 94%; *CaM* + *BenA* = BI/BS 1.00/ 99% BS) and is phylogenetically distant from *A. terreus* (*CaM* = BI/BS = 0.99/<70%; *BenA* = BI/BS 1.00/ 98%; *CaM* + *BenA* = BI/BS 1.00/ 92% BS).

## 3 DISCUSSION

This report highlights a case of a dog on multimodal immunomodulating therapy for IMHA that became coinfected with *A. citrinoterreus* and *C. lunata*. Our case emphasizes the importance of vigilant monitoring for adverse effects related to immunosuppression and opportunistic infections, and it demonstrates the necessity of appropriate and adequate evaluation of lesions that arise after initiation of immunosuppression treatment protocols.

Opportunistic fungal infections in small animals were recently described in a review article by Dedeaux, et al. In that review, it was described how opportunistic mycoses caused by soil saprobes that are typically non‐pathogenic to immunocompetent animals have emerged as clinically relevant causes of disease in patients undergoing immunomodulating therapy for immune‐mediated diseases. The same review also mentions naturally occurring causes of immunocompromise that can predispose patients to opportunistic mycoses, with examples including *Candida* spp. urinary tract infection in diabetic dogs and cats, disseminated aspergillosis and hyalohyphomycosis in German shepherd dogs with suspected familial immunodeficiency, *Pneumocystis* pneumonia in miniature dachshunds with combined variable immunodeficiency, systemic mold infection in young dogs with hereditary cobalamin deficiency, and phaeohyphomycosis in cats with diabetes mellitus, retrovirus infection, or lymphoid neoplasia.^9^, ^10^, ^11^, ^12^, ^13^, ^14^, ^15^, ^16^ Opportunistic mycoses in animals with naturally occurring causes of immunocompromise are rare and generally well‐described. Opportunistic mycoses in patients receiving immunomodulating therapies are more likely to be caused by saprobic organisms that can be more difficult to definitively differentiate.^17^ One report describes an incidence of cutaneous opportunistic fungal infection in dogs receiving immunosuppressive therapy of up to 13%.^18^

*Aspergillus* species are saprobic organisms ubiquitously found in the environment and are opportunistic pathogens. *Aspergillus fumigatus*, *A. flavus*, *A. terreus*, *A. niger*, and *A. deflectus* cause disease in dogs. *A. fumigatus* and *A. flavus* usually cause localized disease in the nasal cavity while *A.terreus* is more commonly associated with systemic aspergillosis in dogs. However, *A. deflectus* and *A. niger* have been reported to cause disease in some dogs.^19^

*A. citrinoterreus* was first described by Guinea et al as a new species of Aspergillus section Terrei in 2015.^5^ It is closely related to *A. terreus* and differs in its colony characteristics and microscopic structure such as the production of abundant yellow diffusible pigment, larger conidiogenous cells and conidia in contrast to the yellow to reddish brown diffusible pigment, smaller conidiogenous cells and smaller conidia produced by *A*. *terreus*. These differences are almost indistinguishable resulting in the misidentification of many isolates as *A. terreus* and consequently misreported. Molecular identification is necessary to distinguish *A. citrinoterreus* from *A. terreus* and from other species in section Terrei. A clinically significant note is that *A. citrinoterreus* was found to be more susceptible to azole antifungals compared to *A. terreus*, but both species still showed clinical resistance to amphotericin B.^5^ Little is known about the geographical distribution of *A. citrinoterreus*. Species in the *Aspergillus* section *Terrei* are reported as common pathogens of invasive aspergillosis in humans.^20^, ^21^, ^22^ Infection with *A. citrinoterreus* has not been reported in the dog. However, since *A. citrinoterreus* has only recently been described, it is possible previous infections of *A. citrinoterreus* may have been misidentified as *A. terreus* infections. In the future, reports of *A. terreus* should have molecular identification performed to differentiate between *A. terreus* and *A. citrinoterreus*, and this may prove beneficial in identification of the prevalence and geographical distribution of *A. citrinoterreus*.

Phaeohyphomycosis is an infrequent infection in both humans and animals, though it has been more frequently reported in recent years in immunosuppressed patients (most commonly in solid organ transplant patients).^17^, ^23^ It is the name given to cutaneous and systemic diseases caused by black molds that develop dark‐walled, septate mycelia in tissue.^24^ These infections are usually cutaneous and acquired traumatically, typically affecting exposed areas of the head or upper limbs, though disseminated cases are reported. Phaeohyphomycosis is an overall term used to describe infection from one of any of over 60 genera of dematiaceous (pigmented) fungi. Agents identified to cause infection in veterinary patients include *Alternaria, Aureobasidium, Bipolaris/Curvularia*, *Cladophialophora*, *Exophiala*, *Fonsecaea*, *Lecythophora*, *Microsphaeropsis*, *Moniliella*, *Mycoleptodiscus* (*Muyocopron*), *Phialophora*, *Ramichloridium*, *Scolecobasidium, Scytalidium*, and *Ulocladium* (*Alternaria*).^17^, ^25^, ^26^, ^27^ These fungi are saprobic, widely‐distributed organisms found in soil, water, and decaying vegetable matter, which are usually non‐pathogenic, but can be devastating to immunocompromised hosts.

The most common clinical manifestations of phaeohyphomycosis in small animals are lesions associated with the digits, pinnae, nasal planum, or the nasal cavity in cats. However, multifocal cutaneous lesions more commonly occur in immunocompromised patients or dogs treated with multiple immunosuppressive agents. Furthermore, the multifocal presentation is even more common when cyclosporine is one of the immunosuppressive agents used.^25^ One report describes an odds ratio of 7.1 for patients diagnosed with cutaneous opportunistic fungal infection that were being treated with cyclosporine.^26^

Phaeohyphomycosis in humans is divided into four forms based on the location of the infection and route of inoculation. These include superficial, cutaneous, subcutaneous, and systemic.^24^ The most common types of phaeohyphomycosis in veterinary medicine are the subcutaneous and systemic forms.^28^ In most of the cases reported in domestic animals, there is no involvement of the epidermis or upper dermis, and traumatic implantation or wound contamination is thought to be the primary mode of infection.^24^

Diagnosis of fungal infections requires microscopic detection of intralesional fungal elements, culture, and often additional diagnostics, such as serology or molecular diagnostics.^29^ The utility of cytology is dependent on sampling and processing techniques, exfoliation of representative populations and extracellular elements, and degree of heterogeneity present within the specimen, which can limit its accuracy. However, cytology can provide rapid, preliminary information that can be helpful in the determination of subsequent diagnostic follow‐up. It should be noted that microscopic evaluation, whether cytologic or histopathologic, is not recommended as a stand‐alone diagnostic tool to determine fungal identification. Culture, isolation, and molecular characterization (PCR, genome sequencing) are required for speciation.^29^ Morphological diagnosis of fungal species is additionally difficult for reasons such as the presence of cryptic species within different genera, the absence of morphological structures that aid in identification, the lack of expertise in fungal identification in clinical microbiology laboratories and the failure for the fungus to grow from clinical specimens. Molecular testing methods using PCR and comparative sequence analysis enable definitive identification of most clinical isolates.^30^ For aspergillosis, diagnosis of fungal infection is achieved through identification of fungal hyphae within tissue specimen or urine or by detection of the fungal cell wall antigen galactomannan in blood or urine as well as DNA sequencing of the isolated fungus from specimens.^31^

Diagnosis of phaeohyphomycosis requires evidence of pigmented fungi in wet mounts or in histologic sections in addition. However, due to similar histologic appearance of many phaeohyphomycotic agents, definitive diagnosis is made by culture and specific identification of the etiologic agent.^15^ Local disease may be cured with excision of diseased tissue, but systemic disease can often be deadly as the disease can often be refractory to therapy.^5^

A comprehensive review of literature reporting disseminated phaeohyphomycosis infections in people identified a total of 72 cases from 1966 to 2002.^32^ In that review, the prognosis is noted to be particularly poor with an overall mortality rate of 79%. Within the cases reported in that review, several treatments were attempted, but none were associated with improved survival. There is one report in veterinary medicine of successful treatment of disseminated cutaneous phaeohyphomycosis in a dog caused by *C. lunata*. That case was treated with a combination of amphotericin B and itraconazole. Interestingly, infection in that case was also suspected to be secondary to immunosuppression with a combination of glucocorticoids and cyclosporine.^33^

The patient in the current report was being treated with immunosuppressive doses of both cyclosporine and prednisone for the management of IMHA. It is likely this therapy caused sufficient immune suppression to induce susceptibility to opportunistic fungal infections. The clinical history provided, in combination with laboratory data, and the gross and microscopic disease, suggest the route of infection was likely through the cutaneous wound followed by hematogenous spread to the previously listed organs. Identification of *Aspergillus* in the pulmonary tissue and microscopic findings of pneumoconiosis and eosinophilic edema are suggestive of inhalation and potential for concurrent direct inhalational disease, and further visceral spread cannot be completely excluded. However, even though thoracic radiographs taken during preliminary work up and additional diagnostics performed ante‐mortem did not show obvious signs of fungal disease aside from a mild monocytosis on initial CBC, it is possible this patient could have been harboring subclinical disease that was either the inciting cause of his immunologic disease or may have been exacerbated during immunosuppressive therapy. Additionally, a fungal susceptibility profile could have been helpful in predicting response to therapy, but due to euthanasia soon after the pleural fluid collection, this was not pursued ante‐mortem. Fungal susceptibility testing may have shown different susceptibility and resistance patterns compared to other *Aspergillus* species, which may have also shown clinical and epidemiological significance.

This report highlights a case of coinfection in a dog with two fungal organisms–*A. citrinoterreus* and *C. lunata–* likely due to multimodal immunomodulating therapy for presumptive non‐associative IMHA. Additionally, this is the first reported case of disseminated *A. citrinoterreus* infection in a dog in North America to the authors' knowledge. Cases of *Aspergillus terreus* species infections are important to note as they may carry a poorer prognosis due to clinical resistance to Amphotericin B and higher mortality rates.^34^ This patient developed a severe disseminated infection of *A. citrinoterreus* in addition to a localized *Curvularia* infection that both led to considerable morbidity and eventually euthanasia. As the two fungal species were collected from different body sites on this patient, this case also highlights the importance of thorough evaluation for possible coinfections in these immunocompromised patients.

## AUTHOR CONTRIBUTIONS

**David Sender:** Investigation; writing – original draft; writing – review and editing. **Benjamin Hulsey:** Writing – original draft. **Connie Cañete‐Gibas:** Methodology; visualization. **Nathan Wiederhold:** Methodology. **Jung Keun Lee:** Methodology. **Abigail Finley:** Methodology. **Catherine Cruz:** Methodology. **Mary E. White:** Investigation; methodology; supervision; writing – review and editing.

## CONFLICT OF INTEREST STATEMENT

The authors declare no conflicts of interests.

## ETHICS STATEMENT

This research did not contain any studies involving animal or human participants, nor did it take place on any private or protected areas. No specific permissions were required for corresponding locations.

## CONSENT

Written informed consent was obtained from the patient to publish this report in accordance with the journal's patient consent policy.

## Data Availability

The data that supports the findings of this study are available in the supplementary material of this article.
